# Clinical Application of ImmunoPET Targeting Checkpoint Inhibitors

**DOI:** 10.3390/cancers15235675

**Published:** 2023-11-30

**Authors:** Elisabetta Maria Abenavoli, Flavia Linguanti, Raffaella Calabretta, Roberto C. Delgado Bolton, Valentina Berti, Egesta Lopci

**Affiliations:** 1 Nuclear Medicine Division, Careggi University Hospital, 50139 Florence, Italy; abenavolie@aou-careggi.toscana.it; 2 Nuclear Medicine Unit, Department of Experimental and Clinical Biomedical Sciences “Mario Serio”, University of Florence, 50134 Florence, Italyvalentina.berti@unifi.it (V.B.); 3 Nuclear Medicine Department, Ospedale San Donato, 52100 Arezzo, Italy; 4 Division of Nuclear Medicine, Department of Biomedical Imaging and Image-Guided Therapy, Medical University of Vienna, 1090 Vienna, Austria; 5 Department of Diagnostic Imaging (Radiology) and Nuclear Medicine, University Hospital San Pedro, Centre for Biomedical Research of La Rioja (CIBIR), 26006 Logroño, Spain; 6 Servicio Cántabro de Salud, 39011 Santander, Spain; 7 Nuclear Medicine Unit, IRCCS—Humanitas Research Hospital, 20089 Rozzano, Italy

**Keywords:** ImmunoPET, PET/CT, CTLA-4, PD-1, PD-L1, checkpoint inhibitors, immunotherapy, cancer

## Abstract

**Simple Summary:**

Cancer treatment with immune checkpoint inhibitors (ICIs) has significantly impacted patients’ management and outcomes. To better assess the benefit of ICIs and reduce the risk of treatment-induced side-effects, new predictive biomarkers have been investigated. From a molecular imaging point of view, several PET tracers have been developed to examine the immune checkpoint (IC) status and the characteristics of the tumor microenvironment (TME). Herein, we present the most relevant data on ImmunoPET-targeting ICs and tumor-infiltrating lymphocytes (TILs), with a main focus on the latest developments in clinical molecular imaging studies of solid tumors.

**Abstract:**

In the last decade, monoclonal antibodies (mAbs) targeting CTLA-4, PD-1, or PD-L1 have been developed and immune checkpoint inhibitors (ICIs) have become the main approach in cancer immunotherapy. However, not all patients benefit from ICI therapy and some are at risk of developing treatment-induced side-effects. These aspects, in parallel with the imaging challenges related to response assessments during immunotherapy, have driven scientific research to the discovery of new predictive biomarkers to individualize patients who could benefit from ICIs. In this context, molecular imaging using PET (positron emission tomography), which allows for whole-body tumor visualization, may be a promising non-invasive method for the determination of patients’ sensitivity to antibody drugs. Several PET tracers, diverse from 2-[^18^F]FDG (or 2-Deoxy-2-[^18^F]fluoroglucose), have been developed to image immune checkpoints (ICs) or key elements of the immune system, although most of them are still in preclinical phases. Herein, we present the current state of the ImmunoPET-targeting of IC proteins with mAbs and antibody fragments, with a main focus on the latest developments in clinical molecular imaging studies of solid tumors. Moreover, given the relevance of the immune system and of tumor-infiltrating lymphocytes in particular in the prediction of the benefit of ICIs, we dedicate a portion of this review to ImmunoPET-targeting T cells.

## 1. Introduction

Cancer cells acquire the ability to escape the immune system through tumor-mediated immune escape mechanisms [1]. Several of them allow cancers to create an immunosuppressive microenvironment, including the downregulation of T-cell cytotoxic responses, through checkpoint-blocking approaches [2]. Under normal physiological conditions, immune checkpoints (ICs) regulate the immune response for the maintenance of self-tolerance and prevention of autoimmunity. The most important proteins involved in checkpoint blocking are cytotoxic T-lymphocyte-associated protein 4 (CTLA-4, CD152) and programmed cell death protein 1 (PD-1, CD279), along with its corresponding ligand, programmed death ligand 1 (PD-L1, CD274) [3]. Based on this concept, monoclonal antibodies (mAbs) targeting CTLA-4, PD-1, or PD-L1 have been developed and immune checkpoint inhibitors (ICIs) have become the main approach in cancer immunotherapy.

Currently, ICIs approved by the US Food and Drug Administration (FDA) for the treatment of various types of cancer include PD-1 inhibitors (nivolumab, pembrolizumab, and cemiplimab), PD-L1 inhibitors (atezolizumab, durvalumab, and avelumab), and a CTLA-4 inhibitor (ipilimumab) [4]. These drugs have resulted in unpreceded outcomes, even in heavily pretreated patients, paving the way for their clinical use in several clinical indications [5]. However, not all patients benefit from ICI therapy and some are at risk of developing treatment-induced side-effects [6]. These aspects, in parallel with the imaging challenges related to response assessments during ICIs, have driven scientific research to the discovery of new predictive biomarkers to individualize patients who could benefit from ICI therapy. In this context, molecular imaging using PET (positron emission tomography), which allows for whole-body tumor visualization, may be a promising non-invasive method for the determination of patients’ sensitivity to antibody drugs [7,8,9,10]. Several PET tracers, diverse from 2-[^18^F]FDG (or 2-Deoxy-2-[^18^F]fluoroglucose), have been developed to image immune checkpoints (ICs) or key elements of the immune system, although most of them are still in preclinical phases [11].

Herein, we present the current state of the ImmunoPET-targeting of IC proteins with mAbs and antibody fragments (i.e., CTLA-4, PD-1, and PD-L1 and its ligand), with a main focus on the latest developments in clinical molecular imaging studies of solid tumors. Moreover, given the relevance of the immune system and of tumor-infiltrating lymphocytes in particular in the prediction of the benefit of ICIs, we dedicate a portion of this review to ImmunoPET-targeting T cells.

## 2. CTLA-4

**Figure 1:**
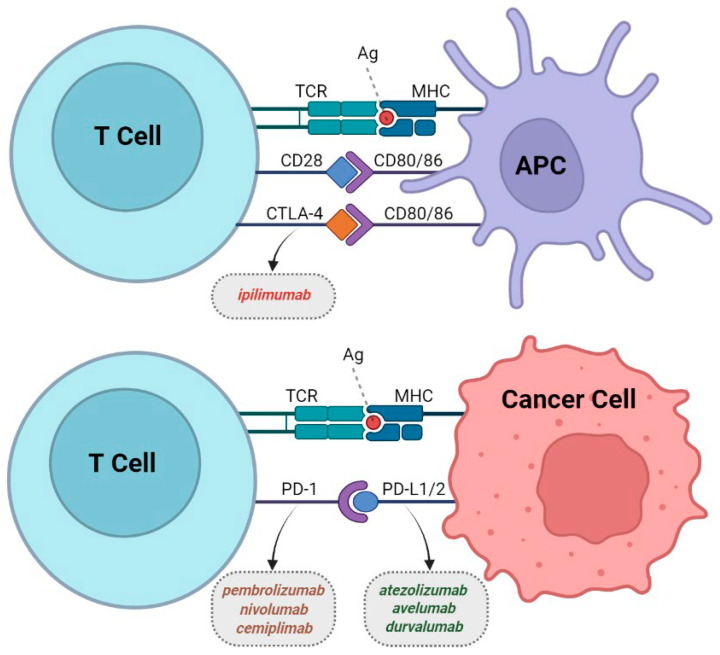
Overview of the immune-checkpoint (IC) targets and classes of IC inhibitors (ICIs). Reproduced from Basudan AM et al. [12] published under a Creative Commons Attribution 4.0 International License http://creativecommons.org/licenses/by/4.0/. accessed 30 November 2023 Notes: CTLA-4 (through the interaction with its ligands B7-1/CD80 and B7-2/CD86) or PD-1 (via binding to its ligand PD-L1) trigger inhibitory signals to attenuate T-cell immune response. These T-cell receptor targets provide rationale for the use of CPIs such as anti-CTLA-4, PD-1, and PD-L1, which are illustrated with dotted-border boxes to increase immune response and kill tumor cells. CTLA-4: Cytotoxic T-lymphocyte-associated protein 4; PD-1/PD-L1: Programmed cell death protein-1 and its ligand-1, respectively; APC: Antigen-presenting cell; Ag: Antigen; TCR: T-cell receptor; MHC: Major histocompatibility complex (Figure was designed with BioRender.com, https://help.biorender.com/en/articles/3619405-how-do-i-cite-biorender, accessed on 7 November 2022).

Cytotoxic T-lymphocyte-associated protein 4, or CTLA-4 (Figure 1), is a transmembrane receptor expressed in activated T cells that binds CD80 or CD86 (B7 ligands), both of which are expressed on the surface of antigen-presenting cells (APCs) and produce inhibitory signals to diminish the immune response. CTLA-4 binds B7 ligands with more avidity than CD28, which normally produces positive signals. In the early stages of carcinogenesis, CTLA-4 can reduce T-cell activation and allow cancer proliferation. By blocking the co-inhibitory receptor CTLA-4 using “checkpoint-blocking” antibodies, T cells can create a cytotoxic immune response in the tumor microenvironment (TME) [12,13,14].

Ipilimumab (Yervoy) was the first ICI antibody approved in 2011 for advanced melanoma and showed a significant improvement in overall survival [15]. Yervoy has also proved to be beneficial when associated with other ICIs in patients with advanced renal cell carcinoma, non-small cell lung cancer (NSCLC), malignant pleural mesothelioma, cancer of the colon or rectum, and squamous esophageal cancer [16].

Currently, only one clinical trial (NCT03313323) is investigating CTLA-4 PET/CT imaging with [^89^Zr]Zr-ipilimumab in patients with melanoma (Table 1). Scans are performed at 2 h, 72 h, and 144 h after the administration of [^89^Zr]-labeled ipilimumab, which is injected within 2 h after the first ipilimumab dose and after 3 weeks for the second treatment cycle, in order to evaluate its potential role as a predictive biomarker for therapy. The study is ongoing; thus, only preliminary data related to the first three patients are available. The data so far show the ability of the tracer to visualize and quantify the amount of ipilimumab uptake in the tumors [17].

In the last years, three preclinical studies have tested an anti-CTLA-4 mAb labelled with [^64^Cu] to visualize CTLA-4 levels in tumor tissues. Firstly, in 2014, Higashikawa et al. developed a molecular imaging probe that targeted CTLA-4 and examined its utility in mice bearing a CTLA-4-expressing CT26 tumor. They demonstrated a significant accumulation of the [^64^Cu]Cu-DOTA-anti-CTLA-4 mAb in the CT26 tumor, suggesting its potential as a non-invasive method to evaluate CTLA-4 expression [18]. Three years later, the biodistribution of [^64^Cu]Cu-DOTA-ipilimumab in mice bearing lung cancer CTLA-4-expressing tumor xenografts was evaluated, demonstrating how the tumor-tracer uptake in vivo could correlate with the CTLA-4 expression in tumor cells. The authors firstly demonstrated in vitro the abundance of CTLA-4 in three NSCLC cell lines, which was subsequently confirmed by [^64^Cu]Cu-DOTA-ipilimumab PET imaging [19].

In 2019, the same authors tested two other tracers radiolabeled with [^64^Cu]-targeting CTLA-4—the full ipilimumab antibody and a F(ab’)2 fragment, respectively—to localize CTLA-4+ tissues in humanized mouse models grafted with peripheral blood lymphocytes. Ipilimumab provided a higher absolute uptake in the target organs compared with the ipilimumab-F(ab’)2 fragment, although the latter allowed higher contrast imaging at earlier timepoints but with lower absolute uptake values. This study pointed out the major differences between full-length antibodies, i.e., mAbs, and antibody fragments [11]. The former were characterized by slow blood clearance and a less optimal target-to-background ratio, and were commonly labeled with long-lived radionuclides requiring repeated imaging after administration. Despite the potential advantages mentioned above, both tracers were able to localize CTLA-4+ tissues [20].

## 3. PD-1/PD-L1

Programmed death ligand 1, or PD-1, is another transmembrane receptor. It belongs to the family of CD28/CTLA-4 immunoglobulin antibodies, whose expression is induced after activation in CD4+ and CD8+ T cells, B cells, and monocytes [21]. This receptor has two ligands, B7-H1 (PD-L1) and B7-DC (PD-L2), even though PD-L1 is present in more cells compared with PD-L2 (Figure 1). Hence, it has a more predominant role in tumor cells [22]. The interaction between PD-1 and its ligands suppresses the activation of antigen-specific T cells in the TME and the lymph nodes; this mechanism is used by cancer-cell-expressing PD-L1 to evade immune surveillance [23].

In 2014, nivolumab was the first anti-PD-1 checkpoint inhibitor approved for the treatment of melanoma and, in the following years, for many other cancer types [24]. Consequently, it was also the first IC target to be radiolabeled and used with ImmunoPET to evaluate its role as a predictive biomarker.

The first in-human PET imaging research using [^89^Zr]Zr-nivolumab was published by Niemeijer et al. in 2018 [25]. The authors analyzed 13 patients with advanced NSCLC prior to treatment with nivolumab. Firstly, they showed its safety and feasibility in a clinical setting and found the optimal timepoint of PET imaging to be 5–7 days post-injection. Their most interesting finding, however, was the correlation between [^89^Zr]Zr-nivolumab uptake and PD-1-positive tumor-infiltrating immune cells measured by immunohistochemistry (IHC), suggesting its potential role in the non-invasive quantification of PD-1 statuses. Moreover, they demonstrated the correlation between tumor-tracer uptake and the therapy response, showing that responding tumors had a higher SUVpeak than non-responding ones [25].

Pembrolizumab is another PD-1 mAb; it has been radiolabeled with [^89^Zr] to assess tumor uptake and whole-body biodistribution before treatment with the anti-PD-1 antibody as well as to explore its relationship with patient outcomes [26,27]. In the first study by Kok et al., [^89^Zr]Zr-pembrolizumab was analyzed in 18 patients with locally advanced or metastatic melanomas and NSCLC. For the study, major lymphoid sites were also visualized using [^89^Zr]Zr-pembrolizumab PET imaging, including the spleen, Waldeyer’s ring, normal lymph nodes, and sites of inflammation. Among the tumor sites, lymph-node metastases showed the highest tracer uptake. With respect to the outcome, the authors showed that patients with a higher [^89^Zr]Zr-pembrolizumab uptake had longer progression-free survival (PFS) and overall survival (OS) than patients with a lower uptake.

Subsequently, Niemeijer et al. analyzed [^89^Zr]Zr-pembrolizumab biodistribution in 12 patients with advanced stage NSCLC [27]. Although the results did not reach a statistical significance, the findings confirmed that patients responding to pembrolizumab had a higher tracer uptake compared with non-responding ones.

So far, other anti-PD-L1-targeting agents have been approved (i.e., atezolizumab, durvalumab, and avelumab), with two of them being radiolabeled and evaluated for imaging in a clinical setting.

Atezolizumab was the first to be evaluated by Bensch et al. in 2018 [28]. The authors enrolled 25 patients with locally advanced or metastatic bladder cancer, NSCLC, or triple-negative breast cancer to assess the feasibility of imaging with [^89^Zr]Zr-atezolizumab and test its potential role for a response prediction to atezolizumab therapy. A high and heterogeneous tumor uptake was documented within and between lesions in all metastatic sites. The authors also demonstrated a significant correlation between the tumor uptake and treatment response as well as PFS and OS [28].

Currently, two separate clinical trials are analyzing [^89^Zr]Zr-labeled atezolizumab for imaging, either locally advanced or metastatic renal cell carcinoma (RCC) (NCT04006522) or metastatic triple-negative breast cancer (MIMIR-mTNBC) (NCT05742269). In both cases, a correlation between the imaging findings and PD-L1 immunohistochemical (IHC) analyses will be performed, aiming to either predict the benefit of ICIs or to select patients with mTNBC that would benefit from the addition of atezolizumab to a chemotherapy backbone.

In 2017, following the results of the PACIFIC trial, another ICI-targeting PD-L1—i.e., durvalumab—was registered for stage III NSCLC patients treated with chemoradiotherapy [29]. Subsequently, two studies have been published so far on the use of [^89^Zr]Zr-durvalumab in cancer imaging. The first one was performed using patients with NSCLC who were eligible for ICI therapy and who underwent [^89^Zr]Zr-durvalumab PET to analyze PD-L1 expression in the tumor sites. The biodistribution of [^89^Zr]Zr-durvalumab and its heterogeneous tumor uptake within and between patients were comparable with the results observed in previous studies [28]. No significant correlation between the treatment response and tracer uptake, as measured by SUVpeak, was proven. However, the authors demonstrated the specificity and stability of the tracer by performing two imaging acquisitions, either with or without a therapeutic predose of unlabeled durvalumab. The imaging series without the unlabeled predose identified a higher number of tumor lesions than the imaging series with the predose [30].

Recently, Verhoeff et al. performed a prospective multicenter phase I–II study (NCT03829007) evaluating the safety and feasibility of [^89^Zr]Zr-DFO-durvalumab PET imaging in 33 patients with recurrent or metastatic squamous cell carcinomas of the head and neck (SCCHN) before monotherapy with durvalumab. In this study, no correlation between the tracer uptake, as measured with SUVpeak, and the treatment response or PD-L1 IHC was found [31]. More promising results are expected from another multicenter clinical trial conducted in Australia (ACTRN12621000171819), which is investigating the role of [^89^Zr]Zr-durvalumab PET in characterizing PD-L1 expression in patients with NSCLC treated with chemoradiotherapy [32].

**Figure 2:**
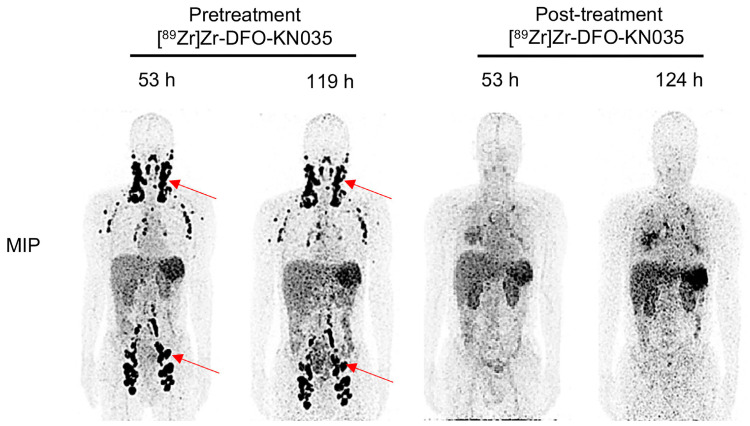
MIP (maximal intensity projection) images of [^89^Zr]Zr-DFO-KN035 PET/CT of a PD-L1-positive (TPS = 30%) patient acquired using ImmunoPET before (53 and 119 h) and after (53 and 124 h) a combined anti-PD-1 therapy. Red arrows show the locations of the primary tumor and metastatic focus sites. Adapted from He et al. [33]. Published under a Creative Commons Attribution 4.0 International License http://creativecommons.org/licenses/by/4.0/ accessed 21 November 2023.

More interesting results were reported recently regarding [^89^Zr]Zr-DFO-KN035 PET/CT [33], a new tracer targeting KN035, which is an anti-PD-L1 monodomain antibody drug for tumor immunotherapy, in clinical research and as a subcutaneous injection [34,35]. In the study, 12 patients with solid malignancies were enrolled and scanned 24, 56, and 120 h after a [^89^Zr]Zr-DFO-KN035 injection and a pretreatment PET, while in 3 patients, a post-treatment evaluation was conducted with images 55 and 120 h after an injection. The statistical data of [^89^Zr]Zr-DFO-KN035 PET showed that the average radioactive uptakes (SUVmax) of multiple tumor foci significantly decreased after the anti-tumor treatment (Figure 2).

As previously mentioned, despite their high specificity and affinity, radiolabeled mAbs show some important limitations related to their large molecular size [36], which dilates the time of biodistribution and makes it necessary for them to be labeled with long-lived isotopes such as [^89^Zr]. For such reasons, imaging with radiolabeled mAbs requires several days of tracer clearance from the circulation to obtain PET images with adequate contrast. To overcome these limitations, radiolabeled antibody fragments are being developed for ImmunoPET.

In 2018, Niemeijer et al. [25] published the first in-human PET imaging study using [^18^F]F-BMS-986192, a 12 kDa adnectin-based human PD-L1-targeting tracer. The authors investigated [^18^F]F-BMS986192 PET prior to treatment with nivolumab in 12 patients with advanced NSCLC. Due to its rapid tissue penetration, it could be radiolabel with shorter-lived isotopes such as [^68^Ga] or [^18^F]. In this study, [^18^F]F-BMS986192 uptake in the tumor lesions correlated with the tumor PD-L1 expression measured by IHC, while a correlation between SUVpeak and the response to nivolumab treatment was also found. More recently, 9 patients with advanced NSCLC who were eligible for nivolumab were exposed to a dynamic [^18^F]F-BMS-986192 PET to quantify the tumor uptake. The authors concluded that SUV at 60 min post-injection, normalized for body weight, could be considered to be an accurate parameter to quantify uptake in tumor lesions [37].

The same molecule used for PET imaging has been subjected to other two clinical trials (Table 1). The first (NeoNivo) focused on combining [^18^F]F-BMS986192 and 2-[^18^F]FDG PET/CT for a response prediction to neoadjuvant nivolumab therapy in oral cancer (NCT03843515), while the most recent trial (NCT05533086) aimed to investigate [^68^Ga]Ga-BMS986192 PET imaging in NSCLC patients undergoing immunotherapy.

In 2022, Zhou et al. [38] evaluated another small-peptide PET tracer targeting PD-L1 ([^68^Ga]Ga-NOTA-WL12) in patients with advanced NSCLC. WL12 is a 14-amino-acid circular peptide engineered to bind PD-L1 with high affinity [39]. Due to its clearance, primarily through the hepatobiliary system, the [^68^Ga]Ga-NOTA-WL12 biodistribution showed a high uptake in the small intestine and liver. Its rapid radioactivity clearance from the blood pool and muscles resulted in high tumor/blood-pool ratios instead. As expected, [^68^Ga]Ga-NOTA-WL12 uptake correlated with the PD-L1 levels detected by IHC. In contrast with previous antibodies radiolabeled with [^89^Zr] with long physical half-lives and circulation times, small-molecule radiotracers such as [^68^Ga]Ga-NOTA-WL12 provide a direct measure of the PD-L1 status within hours of tracer administration.

## 4. Tumor-Infiltrating Lymphocytes

It is widely recognized nowadays that the success of ICIs may be influenced by various mechanisms related to the tumor or to the host, among which the TME plays a central role [40]. Among all components of the TME, the prior recruitment of tumor-infiltrating lymphocytes (TILs), particularly CD8+ cytotoxic T-lymphocytes, placed intratumorally and along tumor margins can impact the outcome of checkpoint blockades [41]. Thus, a non-invasive method of visualizing tumor-infiltrating CD8+ T cells may play a central role in identifying patients with tumors likely to be resistant to immunotherapy as well as in understanding immune-related adverse events resulting from immunotherapy.

**Figure 3:**
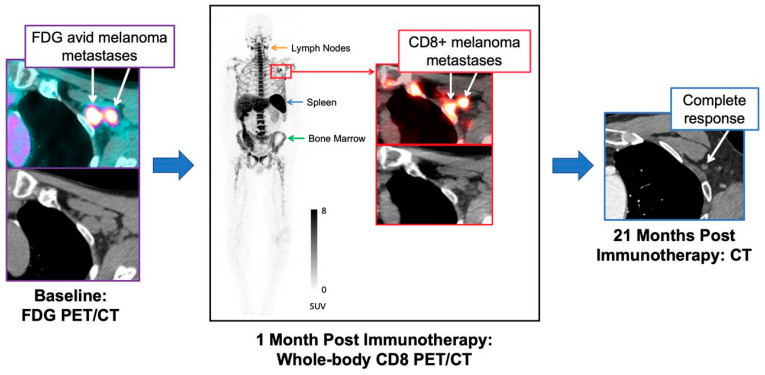
Example of a 71-year-old man with locally advanced stage III melanoma treated with pembrolizumab. Baseline CT and fused 2-[^18^F]FDG PET/CT images (left) demonstrate 2 FDG-avid metastases in left axilla (SUVmax = 10.0, medial node; SUVMAX = 7.6, lateral node). MIP (maximal intensity projection), CT, and fused CD8 PET/CT images (middle) obtained 28 d after start of immunotherapy demonstrate increased tracer activity in both metastases (SUVmax = 9.5, medial node; SUVmax = 10.0, lateral node), suggestive of tumor infiltration by CD8+ T cells. Follow-up imaging with contrast-enhanced CT (right) demonstrated complete response to therapy. Reproduced from Farwell et al. [42] for non-commercial use.

Farwell et al. [42] demonstrated, in phase 1 of the first in-human PET imaging research, the safety and possible predictive value of a [^89^Zr]Zr-labeled anti-CD8 mini-body in 15 patients with metastatic solid tumors (melanoma, NSCLC, and hepatocellular carcinoma) treated with immunotherapy (Figure 3). Before them, Pandit-Taskar et al. in 2020 [43] showed a preliminary report on an evaluation of [^89^Zr]Zr-Df-IAB22M2C imaging in 6 patients with solid tumors who were either undergoing or likely to undergo immunotherapy. In their study, CD8 PET imaging with [^89^Zr]Zr-Df-IAB22M2C resulted in the safe and accurate visualization of the whole-body biodistribution of CD8 leukocytes in tumors and CD8-rich tissues. The maximum uptake was at 24–48 h after the injection, with low background activity in CD8-poor tissues. Farwell et al. [42] confirmed the previous results and although the study was not designed to correlate the tumor uptake with response therapy, they evaluated CD8 PET scans after the initiation of immunotherapy in 3 patients with a good response to therapy at the clinical follow-up. Their CD8 PET scans demonstrated a high [^89^Zr]Zr-Df-IAB22M2C tumor uptake, suggesting that the presence of CD8 tumor-infiltrating lymphocytes correlated with the subsequent therapy response. More data are expected from this radiotracer; since it has been under investigation, a phase II clinical trial has also analyzed different solid tumors (NCT03802123).

Recently, Kist de Ruijter et al. [44] published the results of another phase 1/2 trial (NCT04029181) investigating the biodistribution of a [^89^Zr]Zr-labeled antibody (^89^ZED88082A) targeting CD8 T cells in 38 patients with solid tumors. The study required PET imaging before starting ICI therapy and after ~30 days. The authors confirmed the ability of ^89^ZED88082A PET to assess whole-body CD8+ T-cell distribution, which was not obtainable from a single-lesion biopsy, and showed that the tracer uptake in the tumor lesions correlated with CD8 presence using IHC and autoradiography signals in those same lesions. They also demonstrated that a high ^89^ZED88082A tumor uptake at the baseline was associated with a better OS, pointing out the potential of CD8 imaging as a predictive biomarker to personalize treatments [44].

Subsequently, small alternative antibody formats with fast targeting and high signal-to-background ratios have been developed such as [^64^Cu]Cu-labeled CD8-targeted (IAB22M2C) [45] and [^68^Ga]Ga-NODAGA-SNA006. Wang et al. preliminarily evaluated the distribution and pharmacokinetics of [^68^Ga]Ga-NODAGA-SNA006 in three volunteers with cancer, documenting a high correlation between tumor CD8 expression and [^68^Ga]Ga-NODAGASNA006 uptake, despite the small sample size [46]. Other ongoing trials (Table 1) targeting CD8 are utilizing either [^89^Zr]Zr-Df-Crefmirlimab in stage III melanoma patients (NCT05289193), or [^18^F]F-GEH200521 in irresectable or metastatic solid tumors, or local and resectable head and neck squamous cell carcinomas.

Another important field of application for ImmunoPET is the evaluation of tumor responses to immunotherapies at an early stage. Alternative PET biomarkers such as those targeting granzyme B were recently evaluated by Zhou et al. [47] in a preliminary clinical trial. Granzyme B is a serine protease released by cytotoxic T cells at the end of anti-tumor immune pathways, leading to tumor cell death. Therefore, Granzyme B PET may facilitate the direct visualization of those immune cells killing tumor cells, suggesting its predictive potential for a response to immunotherapy. In the above-mentioned study, patients underwent 2-[^18^F]FDG and [^68^Ga]Ga-grazytracer PET imaging within 1 week of treatment completion; those patients with a positive [^68^Ga]Ga-grazytracer PET showed better responses than those with negative scans. Also, a potential complementary role with 2-[^18^F]FDG PET was reported [47].

Granzyme B is also a target for another tracer under investigation, [^64^Cu]Cu-GRIP B (NCT05888532), or granzyme-targeting restricted-interaction peptide, which is specific to family member B (GRIP B) in advanced genitourinary malignancies (prostate cancer, renal cancer, and urethral cancer) (Table 1). Earlier, Zhao et al. [48] performed a proof-of-concept study assessing the tracer in three syngeneic mouse cancer models to detect Granzyme B in T cells activated with ICIs. As for other studies [49,50] with a different granzyme B tracer, i.e., [^68^Ga]Ga-NOTA-GZP, quantitative measures resulted in a useful predictive biomarker for the assessment of efficacious responses to immunotherapy.

Patient or therapy selection and response assessments to immune-priming therapies can also be investigated using the fluorinated arabinosyl guanine analog, [^18^F]F-AraG. [^18^F]F-AraG was developed as a PET agent to image activated T cells and was useful for the evaluation of both inter- and intrapatient heterogeneity in the immune response to therapy. Furthermore, a change in the [^18^F]F-AraG signal predicted the clinical outcome, as demonstrated in a recent AI-assisted whole-body evaluation of four SCCHN patients who were imaged using [^18^F]F-AraG before and 2–3 weeks after a single dose of an anti-PD-1 antibody. Patients with areas of stable or increasing signals post-therapy had a longer survival than patients where the signal disappeared or decreased post-therapy, indicative of the lack of T-cell activation [51]. An ongoing phase 2 trial (NCT04401995) is further analyzing tracers in melanoma patients treated with the TLR9 agonist vidutolimod (CMP-001) in combination with nivolumab vs. nivolumab alone. Several other trials are also assessing the role of [^18^F]F-AraG PET in NSCLC (NCT04726215, NCT04524195, NCT05157659, and NCT05701176).

Finally, other promising PET tracers for the assessment of immunotherapy responses have been analyzed (Table 1), although, in this context, new applications for already-known radiopharmaceuticals can be found as well. This is the case for the deoxythymidine analog [^18^F]F-FLT. [^18^F]F-FLT is a cell proliferation tracer, which is normally used to evaluate the tumor response after chemoradiotherapy. [^18^F]F-FLT accumulates both in proliferating tumor and immune cells in proportion to the activity of thymidine kinase 1 (TK1), proving potentially useful for tumor response assessments to immunotherapy, as demonstrated by Ribas et al. in patients with metastatic melanomas after a CTLA-4 blockade [52].

## 5. Conclusions and Future Directions

Cancer immunotherapy has revolutionized cancer treatment, but has also raised the need for non-invasive methods to evaluate its efficacy, to better identify patients that could benefit from it, and to optimally evaluate therapy responses. Immune checkpoint assessments using IHC represent the gold standard for patient stratification, although the procedure presents multiple drawbacks. In this review, we focused on the clinical studies evaluating the role of ImmunoPET to assess whole-body IC biodistribution and quantification as well as its predictive value in patients undergoing immunotherapy. As the success of ICIs is also influenced by the TME, we also analyzed the results of CD8 PET imaging studies and other immune-related tracers to characterize the presence of CD8 tumor-infiltrating tissues and their correlation with therapy responses. Finally, we illustrated the alternative PET biomarkers targeting molecules involved at the end of anti-tumor immune pathways that are able to evaluate tumor responses to immunotherapies at an early stage.

The potential clinical relevance of ImmunoPET is undeniable because the immune system and the components of the TME are renowned predictive and prognostic factors of treatment benefits and patient outcomes, limited not only to immunotherapy but also to other regimens. Of particular interest is the synergic effect of combining radiation therapy (RT) with immunomodulatory agents and the expected abscopal effect, which can be explained as being mainly based on immune-mediated component activation, leading to cancer-cell death at distant sites from radiation [53]. Furthermore, the complementary use of ImmunoPET with metabolic imaging could maximize the ability to characterize tumor tissues and assess their responsivity to specific treatments, even more emphasized by the advent of texture analyses and artificial intelligence (AI) [54] in image interpretations.

## Figures and Tables

**Table 1:** Summary of the ongoing clinical trials investigating ImmunoPET for cancer imaging in solid tumors treated with ICIs (source: https://clinicaltrials.gov/), accessed up to 21 November 2023.

Identifier Number	Phase	Study Type	Status	Radiotracer	Study Title	Conditions	Country	Last Update
NCT04271436	II	Interventional	Recruiting	[^18^F]F-FLT	Immune Checkpoint Blockade Therapy Using [^18^F]F-FLT PET/CT	Cancer	USA	2023-05
NCT05471271	NA	Interventional	Recruiting	[^18^F]F-AlF-RESCA-IL2	IL-2 PET Imaging in Advanced Solid Tumours	Metastatic solid tumors	The Netherlands	2023-10
NCT04721756	Early Phase I	Interventional	Recruiting	[^18^F]F-LY3546117	Early Clinical Evaluation of [^18^F]F-LY3546117 in Tumor Imaging	Malignant neoplasms	Australia	2022-04
NCT04706715	I; II	Interventional	Recruiting	[^89^Zr]Zr-DFO-REGN3767	LAG3 PET Imaging in Advanced Solid Tumors	Metastatic solid tumors	The Netherlands	2023-02
NCT04029181	I; II	Interventional	Active; not recruiting	Anti-CD8 agent (ZED88082A)	ImmunoPET with an Anti-CD8 Imaging Agent	Metastatic cancer; unresectable malignant neoplasms	The Netherlands	2023-05
NCT04006522	II	Interventional	Recruiting	[^89^Zr]Zr-DFO-Atezolizumab	[^89^Zr]Zr-DFO-Atezolizumab ImmunoPET/CT in Patients with Locally Advanced or Metastatic Renal Cell Carcinoma	Renal cell carcinoma	USA	2023-06
NCT05000372	NA	Observational	Recruiting	[^68^Ga]Ga-grazytracer	[^68^Ga]Ga-grazytracer PET/CT in Subjects with Non-small Cell Lung Cancer or Melanoma	Non-small cell lung cancer; melanoma	China	2023-03
NCT05888532	I; II	Interventional	Recruiting	[^64^Cu]Cu-GRIP B	[^64^Cu]Cu-GRIP B in Patients with Advanced Genitourinary Malignancies	Prostate cancer, renal cancer, and urethral cancer	USA	2023-06
NCT05629689	I	Interventional	Recruiting	[^18^F]F-GEH200521	A Study to Evaluate GEH200520/GEH200521 (18F) Safety and Tolerability When Used for PET Scans in Patients with Solid Tumour Malignancies	Irresectable or metastatic solid tumors or local and resectable head and neck squamous cell carcinomas	The Netherlands	2023-09
NCT04726215	II	Interventional	Recruiting	[^18^F]F AraG	Imaging of T-cell Activation with [^18^F]F-AraG in Advanced Non-Small Cell Lung Cancer	Non-small cell lung cancer	USA	2023-09
NCT04260256	II	Interventional	Recruiting	[^18^F]F AraG	A Study Using [^18^F]F AraG PET to Evaluate Response to Checkpoint Inhibitor Therapy(CkIT) in Patients with Solid Tumors	Advanced solid tumors	USA	2023-10
NCT04524195	I	Interventional	Recruiting	[^18^F]F AraG	PET Imaging with [^18^F]F-AraG in Advanced Non-small Cell Lung Cancer (NSCLC)	Non-small cell lung cancer	USA	2023-01
NCT05157659	NA	Interventional	Recruiting	[^18^F]F AraG	[^18^F]F-AraG PET Imaging to Visualize Tumor Infiltrating T-cell Activation in Non-small Cell Lung Cancer (ATTAIN)	Non-small cell lung cancer	The Netherlands	2023-04
NCT05701176	NA	Interventional	Recruiting	[^18^F]F AraG	A Clinical Imaging Study of the Changes in [^18^F]F-AraG Uptake Following Anti-PD-1 Therapy in Non-small Cell Lung Cancer (SHARP)	Advanced stage non-small cell lung cancer	The Netherlands	2023-01
NCT05533086	NA	Observational	Recruiting	[^68^Ga]Ga-BMS986192	PD-L1 PET Imaging in Patients with Immunotherapy for Non-small Cell Lung Cancer	NSCLC stage IV; PD-L1 gene amplification	China	2023-02
NCT02453984	NA	Interventional	Active; not recruiting	[^89^Zr]Zr-MPDL3280A	MPDL3280A-imaging-IST-UMCG	Locally advanced or metastatic solid tumors irrespective of PD-L1 expression	The Netherlands	2023-01
NCT04401995	II	Interventional	Recruiting	[^18^F]F-AraG	Study of TLR9 Agonist Vidutolimod (CMP-001) in Combination with Nivolumab vs. Nivolumab	Melanoma	USA	2023-02
NCT05289193	II	Interventional	Recruiting	[^89^Zr]Zr-Df-Crefmirlimab	CD8+ T Cell Imaging During Pre-surgery Immunotherapy in People with Melanoma	Melanoma stage III	USA	2023-04
NCT03843515	I	Interventional	Unknown status	[^18^F]FBMS-986192/2-[^18^F]FDG PET	Neoadjuvant Nivolumab for Oral Cancer Combined with FDG and Anti-PD-L1 PET/CT Imaging for Response Prediction (NeoNivo)	Oral cavity squamous cell carcinoma	The Netherlands	2021-10
NCT05742269	NA	Observational	Recruiting	[^89^Zr]Zr-atezolizumab	Molecular PD-L1 PET/CT Imaging with [^89^Zr]Zr-atezolizumab in Metastatic Triple Negative Breast Cancer (MIMIR-mTNBC)	Metastatic triple-negative breast carcinoma	Sweden	2023-10
NCT03313323	II	Interventional	Unknown status	[^89^Zr]Zr-ipilimumab	Uptake and Biodistribution of ^89^Zirconium-labeled Ipilimumab in Ipilimumab Treated Patients with Metastatic Melanoma (Zirconipi)	Melanoma	The Netherlands	2021-04

Notes: NA: not applicable.
